# D-SPECT combined with deep learning predicts obstructive coronary artery disease

**DOI:** 10.3389/fcvm.2026.1908265

**Published:** 2026-08-19

**Authors:** Binwei Guo, Chunxia Xie, Wenyan Liu, Qi Zhang, Zhifang Wu, Sijin Li

**Affiliations:** 1 Department of Nuclear Medicine, First Hospital of Shanxi Medical University, Taiyuan, Shanxi, China; 2 Shanxi Key Laboratory of Molecular Imaging, Shanxi Medical University, Taiyuan, Shanxi, China; 3 Collaborative Innovation Center for Molecular Imaging of Precision Medicine, Shanxi Medical University, Taiyuan, Shanxi, China; 4 Nuclear Medicine Department, Taiyuan People’s Hospital, Taiyuan, Shanxi, China

**Keywords:** coronary flow reserve, deep learning, D-SPECT, myocardial blood flow, obstructive coronary artery disease

## Abstract

**Background:**

Deep learning models trained on dynamic single-photon emission computed tomography myocardial perfusion imaging (D-SPECT MPI) data may enhance the predictive capability of D-SPECT MPI images for obstructive CAD (OCAD).

**Objective:**

This study aimed to evaluate the predictive performance of deep learning algorithm based on D-SPECT MPI for OCAD.

**Methods:**

A total of 92 patients with suspected coronary artery disease were ultimately included in the study. They underwent D-SPECT MPI and coronary angiography within 6 months. The deep learning OCAD prediction model (DL-OCAD) was trained using perfusion and motion images, and its predictive capability for obstructive stenosis was evaluated through a stratified 5-fold cross-validation process. The comparison of the predictive capabilities for OCAD among DL-OCAD, coronary flow reserve (CFR), stress myocardial blood flow (sMBF), stress total perfusion deficit (sTPD), and summed difference score (SSS).

**Results:**

The total of 56 patients (61%) had OCAD, and obstructive lesions were present in 160 of 276 arteries (58%). The overall diagnostic performance of DL-OCAD (AUC, 0.85; 95% CI, 0.75–0.95) was higher than that of sTPD (AUC, 0.64; 95% CI, 0.50–0.78, *P* = 0.044) and SSS (AUC, 0.63; 95% CI, 0.49–0.78, *P* = 0.028). The sensitivity of DL-OCAD was significantly higher than that of sTPD (81.3% vs. 58.2%, *p* = 0.002) and SSS (81.3% vs. 57.3%, *P* = 0.001). The calibration curves of DL-OCAD, CFR, sMBF, sTPD and SSS models were generally in good agreement with the ideal diagonal. Within the clinically meaningful threshold probability range of 0.05 to 0.5, the DL-OCAD, CFR, sMBF, sTPD, and SSS prediction model can obtain a positive net clinical benefit compared with the strategy of not further examining all patients, and the DL-OCAD curve was always better than the CFR, sMBF, sTPD, and SSS curves.

**Conclusion:**

Compared with existing clinical methods, deep learning has potential clinical value in improving the predictive ability of D-SPECT MPI for OCAD. However, its clinical value still needs to be validated in external studies involving multiple centers and large samples.

## 1 Introduction

The special report published in the Journal of the American College of Cardiology provided a comprehensive analysis of the global burden of cardiovascular disease in 2025. The results indicated that cardiovascular disease remains the leading cause of death globally, with 19.2 million deaths in 2023, a significant increase of 47% compared to 13.1 million in 1990. Ischemic heart disease, cerebral hemorrhage, ischemic stroke, and hypertensive heart disease are the primary cardiovascular causes of disability-adjusted life years (1). Ischemic heart disease is a heart disease caused by insufficient blood supply to the heart muscle due to coronary artery disease (CAD) (2). Therefore, it is necessary to identify the risk factors of the disease and take necessary measures for prevention.

Currently, Single-Photon Emission Computed Tomography (SPECT) myocardial perfusion imaging (MPI) is a non-invasive imaging technique for the diagnosis of CAD, which can provide myocardial perfusion and cardiac function assessment (3). SPECT MPI imaging captures the distribution of intravenously administered 99mTc-methoxyisobutylisonitrile (99mTc-MIBI) within the myocardium and its surrounding tissues, which correlates positively with myocardial blood flow perfusion (4). To reduce injection dosage, save time, and enhance image quality, a new generation of cardiac-specific model, Dynamics SPECT (D-SPECT), has been developed based on cadmium zinc telluride (CZT) semiconductor detectors. Compared to traditional SPECT cameras, D-SPECT exhibits a three to five times higher photon sensitivity and a 1.7 to 2.5 times improved spatial resolution (5). The technological breakthrough has reignited interest in the assessment of SPECT myocardial blood flow (MBF) and myocardial blood flow reserve (MFR) in CAD. For instance, Yamamoto et al. (6) found a correlation between MBF and MFR values detected by D-SPECT MPI and NH3-PET. Zhang et al. (7) found that MBF and MFR measured by D-SPECT were confirmed as predictive indicators of adverse events, and simultaneous assessment of the two could achieve the best prognostic prediction.

Deep learning (DL) specifically refers to the application of machine learning to deep neural network models. It has evolved based on statistical machine learning, artificial neural networks, and other algorithmic models, combined with contemporary big datasets and high computing power. The technical characteristic of DL is its ability to automatically extract features, and it can incorporate image measurement into a learning step, processing data in its natural form (8). As a type of artificial neural network algorithm, DL employs more layers than traditional methods, making it particularly suitable for diverse sets of information (9, 10). Arsanjani et al. (11) showed that compared with quantitative perfusion analysis, using ML method improved the accuracy of MPI in diagnosing obstructive CAD (OCAD) (86% vs. 81%; *p* < 0.01). The polar maps based on DL technology has been directly used in the prediction of OCAD Betancur et al. (12). Trained SPECT MPI images of 1,638 patients with deep learning to establish DL model, and concluded that the ability of deep learning model to predict lesions was higher than that of traditional SPECT parameters total perfusion deficit (per patient: 0.80 vs. 0.78; per vessel: 0.76 vs. 0.73: *p* < 0.01). This study will use the new parameters stress MBF(sMBF), coronary flow reserve (CFR) derived from D-SPECT MPI combined with deep learning algorithm to preliminarily explore the prediction performance of OCAD.

## 2 Methods

### 2.1 Study population

Retrospective selection of patients with suspected CAD assessed between October 2023 and December 2025 for D-SPECT MPI imaging. The inclusion and exclusion criteria are as follows. A consecutive cohort of 102 patients presenting with suspected coronary artery disease symptoms underwent stress/rest/CFR D-SPECT MPI. Ten cases were excluded based on exclusion criteria: four did not complete coronary angiography within the specified timeframe, and six received PCI prior to myocardial perfusion imaging. This study has been approved by the Ethics Committee of our hospital (ethics number: 2025k005), and the experimental procedures comply with the guidelines of the Helsinki Declaration. Prior to the examination, all patients were informed of the purpose and procedures of the clinical trial. All patients agreed to this clinical trial and signed informed consent forms.

Inclusion criteria:

1. Clinical symptoms, electrocardiogram, or cardiac enzyme levels suggestive of or indicative of myocardial ischemia, with suspected CAD;
2. Availability of complete D-SPECT MPI results and coronary angiography (CAG) findings within the past 6 months.

Exclusion criteria:

1. Absence of CAG results for assessment of coronary artery stenosis;
2. History of percutaneous coronary intervention (PCI) or coronary artery bypass grafting (CABG) prior to D-SPECT MPI;
3. Presence of structural heart disease, including congenital heart disease, severe valvular heart disease requiring surgical intervention, cardiomyopathy, atrial fibrillation, intracardiac thrombus, cardiac tumor, pericardial disease, ventricular septal defect, or ventricular aneurysm (13).

### 2.2 D-SPECT protocol

Patients must suspend beta-blockers, calcium channel blockers, nitrates, and any caffeine-containing products 24 to 48 h prior to D-SPECT examination. All patients undergo a “one-day” imaging protocol with resting/stress scans using 99mTc-MIBI. The radiotracer is administered via bolus injection through an automatic injector to ensure consistent quality and reproducibility of radiotracer infusion (14). Image acquisition was performed utilizing a D-SPECT cardiac scanner (Spectrum Dynamics, Caesarea, Israel) equipped with nine CZT detectors. Dynamic images were acquired in list mode. For resting imaging, an initial tracer dose of approximately 37 MBq (1 mCi) was administered to position the heart within the field of view and establish the region of interest (ROI). Subsequently, the remaining dose of approximately 148 MBq (4 mCi) was injected to complete a 6-minute resting dynamic scan. Following a 25-minute delay, a 6-minute resting perfusion scan was performed. During stress acquisition, the heart position could be directly localized using residual tracer from the preceding rest scan. The patient was then administered a pharmacological stress agent (adenosine or Regadenoson). Simultaneously, radiotracer injection and a 6-minute dynamic scan were initiated at peak perfusion. Twenty-five minutes later, a 4-minute stress perfusion scan was completed. The entire procedure acquires a complete rest-stress dataset within approximately 75 min for estimating coronary flow reserve (CFR) (15).

### 2.3 D-SPECT image analysis

All analyses were performed using Corridor 4DM software (INVIA Medical Imaging Solutions, Ann Arbor, MI, USA). Data were typically resegmented into 32 frames comprising 3 s  ×   21 frames, 9 s ×  1 frame, 15 s ×  1 frame, 21 s ×  1 frame, 27 s ×  1 frame, and 30 s ×  7 frames. Dynamic imaging acquisitions were reconstructed using the ordered subset expectation maximization (OSEM) algorithm with 4 iterations, generating 32 subsets, applied with the normalized filter of the D-SPECT cardiac scanner (16).

According to the standard myocardial segmentation method recommended by the American Heart Association, the polarity map image is divided into 17 segments. The tracer uptake in each segment is evaluated (17). Each image was independently interpreted by three experienced nuclear medicine specialists using a blinded approach, with any discrepancies resolved through consensus. The five point scale (0 = normal, 1 = vague, 2 = moderate, 3 = severe reduction of radioisotope uptake, 4 = no detectable radioisotope activity in this segment) was applied to conventional visual assessment of stress and rest perfusion images (18). By summing the individual scores of 17 segments under stress and at rest, the summed stress score (SSS) and summed rest score (SRS) are derived (16). Since quantitative target cardiogram has hundreds of myocardial regions, compared with the simplified 17 myocardial segments, its assessment of the severity of myocardial perfusion decline can be directly obtained through continuous abnormal scores, and the estimation of the range of myocardial perfusion decline is more accurate. The assessment result is often referred to as total perfusion defect (TPD) (19). According to resting state and stress state, it can be divided into resting total perfusion defect (rTPD) and stress total perfusion defect (sTPD). Both the quantitative SSS, SRS and the total deficit score are derived from the same quantitative perfusion map (20).

Time-activity curves of the left and right ventricular blood pools serve as input functions for biventricular kinetic modeling analysis. K1 is defined as the product of MBF and the extraction fraction. K1 values are calculated for stress and rest images, while K2 is set to zero. MFR is the ratio of stress to rest K1 values. The polar map is divided into three regions: left anterior descending artery (LAD), left circumflex coronary artery (LCX), and right coronary artery (RCA), with MFR calculated for each region. CFR analysis employs the ROI blood sampling method, achieved by averaging the boxed region within the left ventricular blood pool. Specifically, the short axis is centered on the left ventricle, and the long axis is centered on the basal valvular plane across all time intervals. The ROI size is two pixels wide in the short axis and 30 mm long in the long axis for sampling the left ventricular and left atrial cavities (21).

### 2.4 CAG analysis

CAG examinations were performed according to standard clinical protocols within 6 months of D-SPECT MPI testing. All coronary angiography images were visually interpreted by on-site two cardiologists. A 50% or greater reduction in the lumen diameter of the left main coronary artery (LMA), or a 70% or greater reduction in the lumen diameter of the LDA, LCX, or RCA, was considered the gold standard for obstructive CAD (22).

### 2.5 Deep learning model

**Figure 1:**
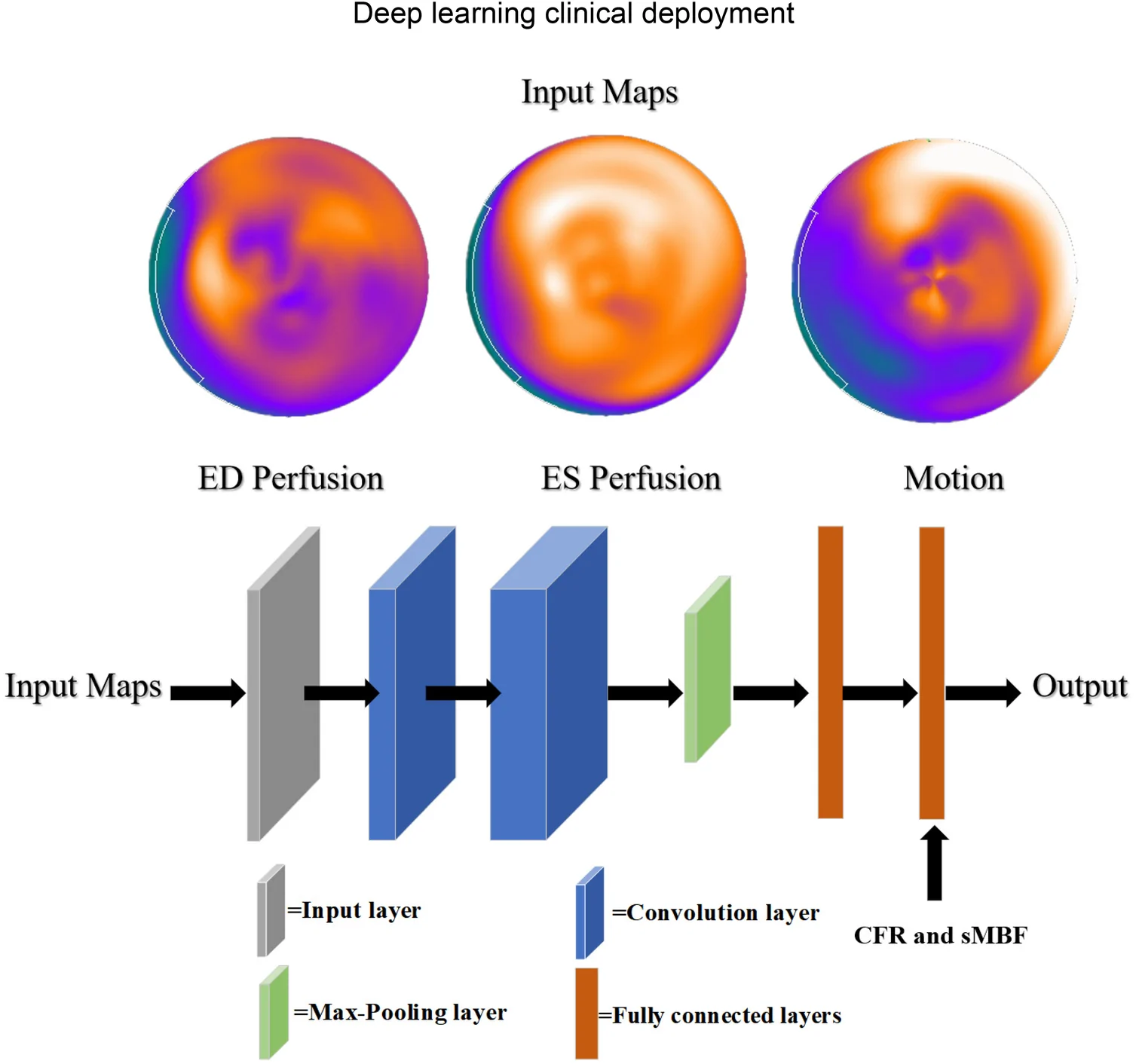
Deep learning clinical deployment. The myocardial perfusion and motion images are inputted into the deep learning model “DL-OCAD”. CFR and sMBF are added to the final fully connected layer, which is used to estimate the probability for each patient and each vessel. ED = end-diastolic volume; ES = end-systolic volume; CFR = coronary flow reserve; sMBF = stress myocardial blood flow.

The deep learning prediction of obstructive coronary artery disease model (DL-OCAD) is trained using perfusion and motion maps, along with CFR and sMBF values. The DL-OCAD is implemented using Python version 3.7 and TensorFlow version 1.14. The overall workflow is as follows (Figure 1):

1. Process perfusion（end-diastolic volume and end-systolic volume）and motion images from the original load and rest scans. Input images of dimension 64 × 64 × 3 are fed into a convolutional-fully connected network. This fully connected network comprises two convolutional layers with 32 and 64 kernels, respectively, followed by a 2 × 2 kernel max pooling layer. The LeakyReLU activation function with a negative slope of 0.2 is used at all layers of the network. To prevent overfitting, the convolution layer is constrained by a probability dropout mechanism of 0.3 and an L2 kernel regularizer (weight coefficient 0.01).
2. During the training process, the potential features extracted from the convolution layer are flattened, and two fully connected layers consisting of 128 hidden units are input. Each layer adopts L2 regularization and sets a dropout rate of 0.3. sMBF, CFR and other numerical image features are finally spliced with the output results of the full connection layer.
3. DL-OCAD outputs a 1 × 4 vector containing probability vectors for left anterior descending, left circumflex, and right coronary arteries, along with the patient's overall occlusive coronary artery disease probability, ranging from 0 to 1 (23).
4. Repeatability testing: The dataset of all patients was randomly divided into 3 separate parts or folds. Each part or fold contained the same proportion of CAD patients. Each training set consisted of 2 folds (accounting for 66% of the total dataset), while the remaining folds (comprising 33% of the complete dataset) were used for testing. The method of 5 repeated tests maximized the data available for training.
5. The Sigmoid activation function is adopted for prediction. The model utilizes the cross-entropy loss function and the Adam optimizer, with an initial learning rate of 0.001 and a batch size of 128. Hyperparameters such as stopping criteria and learning rate were tuned using 5-fold internal cross-validation.

### 2.6 Statistical analysis

Deviations from normal distribution in continuous variables were expressed as median and interquartile range. Continuous variables were analyzed using the Mann–Whitney U test. Categorical data are presented as frequencies and percentages, analyzed using chi-square test. The Cohen's Kappa was used to analyze the consistency of the results between the two observers. Logistic regression was used to analyze the relationship between D-SPECT MPI parameters and OCAD. The area under the receiver operating characteristic curve (ROC) was used to compare the diagnostic performance of DL-OCAD with other diagnostic methods. The diagnostic integrity of ROC curve was compared by using Delong test and Bonferroni multiple correction test. The sensitivity and specificity of the best diagnostic performance were found through the principle of maximizing the Youden index, and were compared by chi-square test. The calibration of univariate logistic regression models were evaluated through Hosmer-Lemeshow decile grouping. For DL-OCAD, Python was used to calculate the same decile grouping strategy to generate calibration curves, in order to achieve consistent visual comparison between all predictive factors. Decision curve analysis was used to construct a univariate logistic regression model with traditional parameters to generate predicted disease probabilities. For DL-OCAD, the continuous prediction probability comes directly from model inference, by using Python to calculate the net clinical benefit within a threshold probability range of 0 to 1. A two-tailed *P* value < 0.05 was considered statistically significant. Statistical analysis was performed using Python version 3.7, and images were generated using graphpad prism 10.1.2.

## 3 Result

### 3.1 Clinical and CAG characteristics

Ultimately, 92 patients were included. Patients were classified into obstructive and non-obstructive groups based on obstructive CAD, defined as ≥50% stenosis of the left main coronary artery or ≥70% stenosis of other arteries. The OCAD group was older [60.5 (50.0–69.0) vs. 64.0 (53.0–74.0), *p* = 0.006] and had a higher proportion of men (38 (67.9%) vs. 16 (44.4%). The total of 56 patients (61%) had obstructive CAD, and obstructive lesions were present in 160 of 276 arteries (58%). The proportion of patients without vascular disease was higher in the non OCAD group [10 cases (27.8%) vs. 0% (0%), *P* < 0.0001], while the proportion of patients with three vessel disease was higher in the OCAD group [6 cases (16.7%) vs. 25 cases (44.6%), *P* = 0.006]. There was no significant difference in the incidence of single vessel disease and double vessel disease between the two groups (12 (33.3%) vs. 13 (23.2%), *P* = 0.287; 8 (22.2%) vs. 16 (28.6%), *P* = 0.499). There were no statistically significant differences in other baseline clinical variables or cardiovascular risk factors. Population characteristics and coronary artery lesion distributions are shown in Table 1.

**Table 1:** Population and coronary artery lesion characteristics.

Characteristic	Non-OCAD (n 36)	OCAD (n 56)	*P*
Age (y)	60.5（50.0–69.0）	64.0（53.0–74.0）	0.006
Male	16 (44.4%)	38 (67.9%)	0.026
BMI, kg/m2	27.8（23.2–29.7）	28.1（23.2–31.2）	0.121
Diabetes mellitus	17 (47.2%)	28 (50.0%)	0.795
Hypertension	19 (52.7%)	25 (44.6%)	0.446
Dyslipidemia	24 (66.7)	39 (69.6%)	0.764
Smoking	19 (52.8%)	36 (64.3%)	0.272
0-vessel disease	10 (27.8%)	0 (0%)	<0.0001
1-vessel disease	12 (33.3%)	13 (23.2%)	0.287
2-vessel disease	8 (22.2%)	16 (28.6%)	0.499
3-vessel disease	6 (16.7%)	25 (44.6%)	0.006
Left main disease＞50%		19 (33.9%)	
LAD disease＞70%		26 (46.4%)	
LCX disease＞70%		28 (50.0%)	
RCA disease＞70%		19 (33.9%)	

Values are median (interquartile range) or frequency (percentage).

OCAD, Obstructive coronary artery disease; Non-OCAD, Non obstructive coronary artery disease; BMI, body mass index; LAD, left anterior descending artery; LCX, left circumflex coronary artery; RCA, right coronary artery.

### 3.2 Consistency and logistic regression analysis

The two on-site cardiologists had good consistency in the interpretation of OCAD (Cohen's Kappa=0.685，*P* = 0.0003). As shown in Table 2, Simple logistic regression demonstrated that CFR (*β* = −1.894, OR = 0.150, 95% CI: 0.034–0.465, *P* = 0.0003), sMBF (*β* = −1.432, OR = 0.239, 95% CI: 0.068–0.638, *P* = 0.0029), sTPD (*β* = 0.379, OR = 1.461, 95% CI: 1.056–2.230, *P* = 0.017) and SSS (*β* = 0.396, OR = 1.486, 95% CI: 1.061–2.289, *P* = 0.018) were significantly associated with OCAD (Table 2A). The above four significant variables were included in the multivariate regression model. CFR (*β* = −1.519, OR = 0.219, 95% CI: 0.044–0.846, *P* = 0.041) was still correlated with OCAD and may be a potential independent protective factor (Table 2B). Multiple logistic regression model calibration was evaluated by Hosmer-Lemeshow test; The statistical results showed a good fit (*p* = 0.349).

**Table 2A:** Simple logistic regression between D-SPECT MPI parameters and OCAD.

D-SPECT parameters	*β*	OR	95% CI	likelihood ratio test	*P*
CFR	−1.894	0.150	0.034–0.465	13.050	0.0003
sMBF	−1.432	0.239	0.068–0.638	8.882	0.0029
rMBF	0.347	1.414	0.122–17.220	0.078	0.781
sTPD	0.379	1.461	1.056–2.230	5.712	0.017
rTPD	0.200	1.221	0.861–1.799	1.224	0.269
SSS	0.396	1.486	1.061–2.289	5.638	0.018
SRS	0.266	1.304	0.897–1.964	1.904	0.168

**Table 2B:** Multiple logistic regression analysis of D-SPECT MPI parameters and OCAD.

D-SPECT parameters	β	OR	95% CI	*P*
CFR	−1.519	0.219	0.044–0.846	0.041
sMBF	−0.763	0.4662	0.106–1.795	0.281
sTPD	0.189	1.209	0.737–2.212	0.488
SSS	0.125	1.133	0.669–1.988	0.646

OR, Odds ratio; 95% CI, 95%confidence interval; CFR, coronary flow reserve; sMBF, stress myocardial blood flow; rMBF, rest myocardial blood flow; sTPD, stress total perfusion defect; rTPD, rest total perfusion defect; SSS, summed stress score; SRS = summed rest score.

### 3.3 DL-OCAD diagnostic capabilities

**Figure 2:**
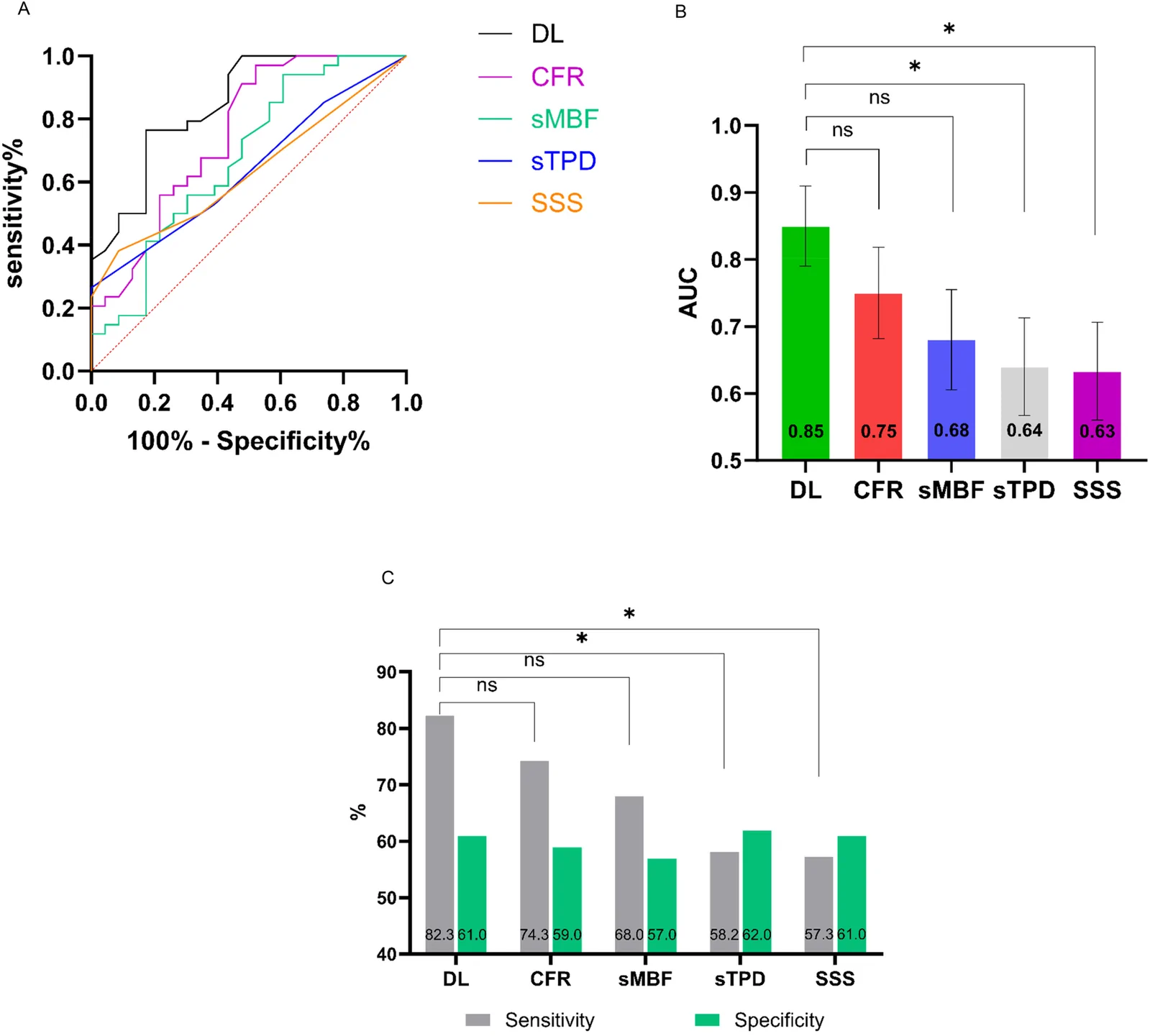
**(A)** ROC between DL-OCAD and D-SPECT MPI index. **(B)** AUC of DL-OCAD and D-SPECT MPI index. **(C)** Comparison of sensitivity and specificity between DL-OCAD and D-SPECT MPI index. ROC = receiver operating characteristic curve; AUC = area under the receiver-operating characteristic curve; DL = deep learning prediction of obstructive coronary artery disease model; CFR = coronary flow reserve; sMBF = stress myocardial blood flow; sTPD = stress total perfusion defect; SSS = summed stress score.

The overall diagnostic performance of DL-OCAD (AUC, 0.85; 95% CI, 0.75–0.95) was higher than that of sTPD (AUC, 0.64; 95% CI, 0.50–0.78, *P* = 0.044) and SSS (AUC, 0.63; 95% CI, 0.49–0.78, *P* = 0.028). However, DL-OCAD (AUC, 0.85; 95% CI, 0.75–0.95) showed no statistically significant difference compared to CFR (AUC, 0.75; 95% CI, 0.62–0.89, *P* = 0.151) or sMBF (AUC, 0.68; 95% CI, 0.53–0.82, *P* = 0.087) (Figures 2A,2B). The highest sensitivity was achieved by DL-OCAD, which was significantly higher than that of sTPD (81.3% vs. 58.2%, *P* = 0.002) and SSS (81.3% vs. 57.3%, *P* = 0.001). There was no significant difference between DL-OCAD and the two indices (CFR, sMBF). The specificity of DL-OCAD was not significantly different from other parameters (Figure 2C).

### 3.4 Calibration and decision curve

**Figure 3:**
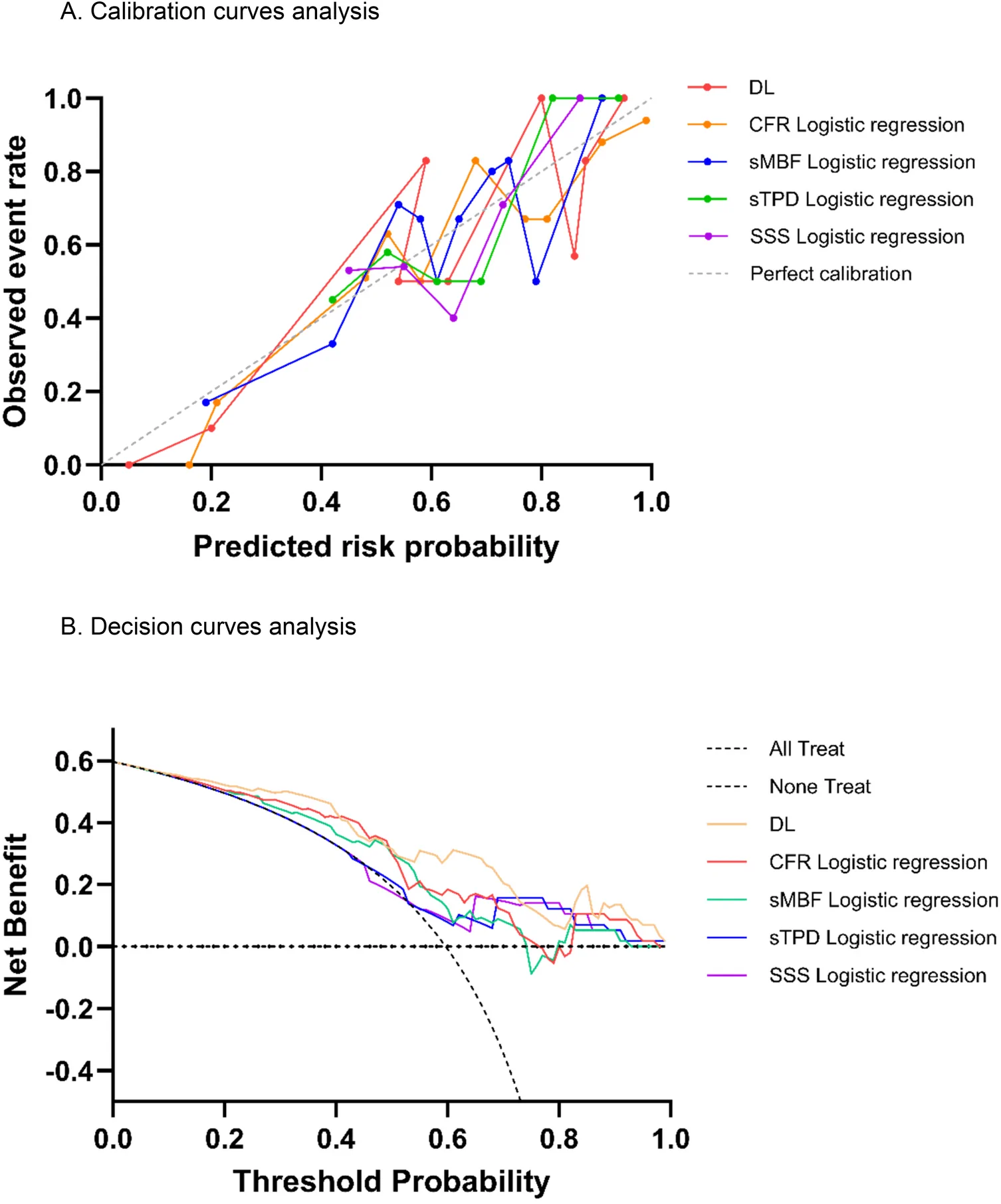
**(A)** calibration curves analysis. **(B)** Decision curves analysis. DL = deep learning prediction of obstructive coronary artery disease model; CFR = coronary flow reserve; sMBF = stress myocardial blood flow; sTPD = stress total perfusion defect; SSS = summed stress score.

Based on the drawn calibration curves, the calibration curves of DL-OCAD, CFR, sMBF, sTPD and SSS models fit well with the ideal 45° diagonal as a whole (Figure 3A). Decision curve analysis was used to evaluate the clinical application value of DL-OCAD, CFR, sMBF, sTPD and SSS in identifying obstructive coronary heart disease. Within the clinically meaningful threshold probability range of 0.05∼0.5, the DL-OCAD, CFR, sMBF, sTPD, and SSS prediction models can achieve positive net clinical benefits compared to the strategy of not receiving further examination for all patients. The DL-OCAD curve consistently outperforms the CFR, sMBF, sTPD and SSS curves in the vast majority of threshold intervals, indicating that it has better net clinical benefits in guiding subsequent examinations (Figure 3B).

## 4 Discussion

This study utilizes deep learning techniques to predict obstructive coronary artery disease (CAD) based on D-SPECT MPI perfusion images and motion images, and compares them with clinically recognized perfusion quantification indicators (including CFR, sMBF, sTPD, and SSS). The research results indicate that the deep learning model combining raw perfusion images with quantitative perfusion images and motion images is superior to traditional CFR, sMBF, sTPD and SSS methods in predicting obstructive lesions. A previous large-scale multicenter study showed that an AI automated obstructive CAD diagnostic system using perfusion quantitative data and clinical measurements through deep learning was superior to the TPD method used alone in predicting obstructive lesions (12). By utilizing D-SPECT to provide CFR and sMBF data for deep learning training, the aim is to improve the predictive ability of obstructive CAD. In this study, we demonstrated that deep learning can improve standard clinical methods. The ability of deep learning to quantify relevant measurement values from raw image data by integrating imaging data will play an important role in cardiovascular medicine. Compared with quantitative perfusion evaluation, the AUC of each patient in the DL-OCAD model is significantly higher, and DL-OCAD has roughly equivalent specificity, showing significantly higher sensitivity. Artificial intelligence can be used to highlight the characteristics of coronary artery stenosis in coronary computed tomography angiography (24) or to emphasize myocardial scarring and fibrosis in cardiac magnetic resonance imaging (25). All these potential applications of interpretable artificial intelligence can aid in improving clinical decision-making for these methods (26). Deep learning can extract potential information from data and help readers by highlighting areas of interest. Similarly, the DL-OCAD model is designed to present and interpret predictions to help doctors interpret. We tried to use the DL-OCAD model to improve the prediction of vascular disease in each vessel of each patient. However, due to the small sample size and sample deviation, the vascular disease ability of this deep learning has not been realized in this study. These studies show that deep learning models have the potential to enhance physicians' ability to interpret, thereby improving the diagnostic level of obstructive CAD.

This preliminary study applying deep learning to D-SPECT MPI has several limitations. First, the degree of CAG was visually interpreted, as quantitative angiography is not routinely performed at many sites. However, we employed a 70% diameter stenosis cutoff based on obstructive CAD diagnosis, which has been demonstrated to better distinguish functionally significant lesions than a 50% cutoff (27). Secondly, we used images from pressure/resting perfusion and polar plots collected at a single location. When training deep learning diagnostic models, pressure/rest perfusion images and polar plots from the same patient can sometimes produce different results. This study cannot predict or specifically analyze the anatomical location of vascular obstruction. A larger training dataset may enhance this ability. Thirdly, This study showed a high incidence of disease: 61% have obstructive coronary artery disease (CAD), which was a population with a high probability of pre screening, limiting its generalizability in screening or low-risk scenarios, which may reduce the effectiveness of predicting OCAD. Fourthly, The small sample size may limit the extrapolation of research conclusions; Using single center queue to carry out internal verification, lack of independent external queue verification; The stability of each prediction model still needs to be further confirmed by external multicenter data. Therefore, this experimental model was currently a hypothetical generative analysis, which was a preliminary exploration of the deep learning model. Prospective external cohort validation will need before deep learning prediction is widely used in clinic.

## 5 Conclusion

Compared with the existing traditional clinical methods, deep learning improves the clinical potential value of the automatic prediction performance of D-SPECT on OCAD. However, it is still necessary to confirm its performance through external large-scale samples and multi center validation.

## Data Availability

The datasets presented in this article are not readily available because the data that support the findings of this study are not openly available due to institutional restrictions and are available from the corresponding author upon reasonable request. Requests to access the datasets should be directed to Sijin Li at lisjnm123@163.com.
